# On the Time Required to Produce Death by a Fatal Dose of Medicinal Hydrocyanic Acid

**Published:** 1847-12

**Authors:** S. C. Sewell

**Affiliations:** Lecturer on Materia Medica, University M’Gill College, etc., etc.; Montreal


*On the time required to produce Death by a fatal dose of Medicinal Hydrocyanic Acid.* By S. C. Sewell, M. D., Lecturer on Materia Medica, University M'Gill College, etc., etc.—My attention has been attracted to this subject in consequence of a fatal case having occurred in my practice lately. A *resumé* of the history of some of the more remarkable instances of fatal, and nearly fatal cases on record, will be necessary to elucidate the interest attached to this point. In the case of the seven Paris epileptics (1828), where a very concentrated acid was used (the half-ounce potion contained 18½ grs. pure acid), some lingered as long as twelve minutes before life was entirely extinct; but the first who swallowed it was dead in three minutes. The first time that the life of a prisoner depended upon a solution of the question under consideration, occurred at the Lancaster Assizes, held in April, 1829, when Freeman, an apothecary's apprentice, was arraigned for the murder of Judith Burwell, his master's servant. She was pregnant by him, and was found one morning dead in her bed. An ounce phial containing three drachms of prussic acid, corked, and wrapt in paper, was found alongside of her. The body was in a composed position, the arms folded over the trunk, and the bedclothes drawn smoothly up to the chin. Had the deceased time to perform all these actions after drinking the poison out the narrow-necked phial? Messrs. Macaulay, Paget, and others, in consequence of experiments performed on the lower animals, decided in the negative. Dr. Christison, in the first edition of his work on Poisons, said that his experiments accorded with theirs; but, in the second, that it was probable that prussic acid frequently took a longer time to act than was generally supposed, and that the probability in this case was that it had done so, and that it had been taken voluntarily by the deceased, because the prisoner had to pass through the room in which his master and mistress slept, to gain access to the girl's room, and must have opened and shut three doors without noise. My opinion is, that she took it voluntarily to produce abortion, for which she had made preparations the night before, and that, if Freeman had anything to do with it, he provided her, for his own purposes, with the poison, telling her that it would cause miscarriage. Mrs. Latten died in twelve minutes from taking a drachm and a half of medicinal acid. In Dr. Geoghegan's case, the patient took two drachms of prussic acid (Dub. Pharm.), and experienced no effect for *two minutes*. He subsequently fell into violent convulsions, and was saved by applying sesquicarbonate of ammonia to the nostrils. In the July number of the London Medical Gazette, is quoted Mr. Godfrey's case of "a man 44 years of age, who, after taking half an ounce of Scheele's acid, walked ten paces to the head of the stairs, descended the steps, seventeen in number, and then proceeded, rather quickly, to a druggist's shop, forty-five paces distant, where he had procured the acid, entering the shop in his usual slow and easy manner, and asking for 'more of that prussic acid,' before he became evidently affected by the poison which he had swallowed. In this instance, at least *five minutes* must have elapsed, from the time of swallowing the poison, before death took place." This case is quoted as introductory to the report of a coroner's inquest, which took place at Worcester on the body of Mr. Benjamin Shepherd. The substance is as follows:—Mr. S. went into Mr. Stringer's (druggist) shop, and purchased ʒij "prussic acid, Scheele's strength, and, asking if any one was in the back room, and being answered in the negative, walked in there, saying to the druggist, "I want a word with you." Stringer followed him within *two minutes*, and found him sitting on the sofa, and the phial of prussic acid empty on the table before him. Stringer said, "good God, Shepherd, you have not been taking that? Deceased replied, smiling, "No, no, it is all right—take no notice—give me your hand old fellow." Witness went up to him and the deceased added, "God bless you—its all right—take no notice." Witness went for Mr. Griffith, surgeon, but, not finding him, returned with Mr. Pierpont, who with witness, tried to administer *ammonia as an antidote* to the prussic acid, and a futile attempt was made to produce vomiting. The stomach pump was sent for, but arrived after death had taken place. Before leaving this case, I must comment upon the means employed to save Mr. Shepherd. Mr. Pierpont and Mr. Stringer should have known that, by administering ammonia, they would have formed the hydrocyanate of ammonia, nearly, if not quite, as energetic a poison as the prussic acid; and that ammonia or the sesquicarbonate applied to the nostrils, acts usefully by stimulating the nervous system, and the heart's action, until the poison has exhausted its violence, and not as an antidote. Secondly, attending on vomiting, and the stomach pump was doubly useless, inasmuch as had they evacuated the stomach, they would have been no nearer saving their patient, and they thereby lost precious time which might have been employed in using more efficacious means. As an antidote, a solution of the sulphate of iron, or a dilution of the Tr. Fer. Mur. would have been as effectual as an antidote can be in a case of poisoning by this acid. The application of chlorine water or sesquicarb. ammonia to the nostrils, and cold affusion to the spine, would have comprised all that is known to be of value in the treatment of such unfortunate cases.

My patient had been for a long time hypochondriacal, and had frequently threatened to destroy himself. During the day of the fatal event, he repeatedly told his relations that he would be dead by nine that night; but, as he had frequently said the same thing, no attention was paid to it. At six in the evening, he purchased an ounce phial of Prussic acid, Scheele's strength, and on his road home, shewed it to several persons, saying that he would soon be dead, and invited them to his funeral. At seven in the evening, he took leave of his friends in a gay, smiling manner, and going up to his room, sent for Mrs. ——, shewed her the poison, and said that he would be dead in two minutes. She snatched at the phial, but he drew it playfully away, turned her out of the room, and locked the door. She, thinking that he was jesting, as he had frequently done the same thing before, went to her own house, next door, which communicated through the yards. About a minute after, he unlocked the door and cried out, "Come to me quick, I am dying." A relative, very much alarmed, called to the servant man in the yard, who ran up stairs and found him lying on his back on the sofa with his legs crossed, insensible, and snoring. In a few moments, Mrs. —— arrived and found him in the same state. I arrived there in twenty minutes. He was then dead, and presented the appearance of profound slumber; the legs crossed, the arms by his side, and eyelids firmly closed. I applied Liq.

Am. Fortissim. (a strength made for portability by manufacturing chemists) to the nostrils, and cold affusion to the occiput and spine. I considered him dead, but employed the remedies in the event of a possibility of there being some remaining sparks of life. The eyes were much more brilliant than during his life, and continued so the next day; the face was livid, and the lips very blue; the muscles were all flaccid, and exhibited no tonicity, except a little in the legs at the end of twenty hours. No sectio was permitted. The phial, containing a drachm of prussic acid, was on a table, ten feet from the sofa, with a wine glass upset and broken alongside, done by the deceased in the hurry of putting it on the table. After having employed my remedies, I applied my nose to the deceased's mouth, but could detect no smell of prussic acid. The remaining acid was thrown out by the servant, so that I could not ascertain its strength; but I feel certain that it was acid of the strength of over three per cent., which is the usual strength of medicinal acid imported into this country; and, since the use of ground glass stoppled phials to put it up in, it always reaches here unimpaired in quality. In the present case, seven drachms of medicinal acid, containing about twenty-one grains of pure acid, were swallowed. The friends think about a minute elapsed before he unlocked the door; but more must have passed, because Mrs. —— had time to go to her own house and busy herself in household affairs before the alarm was given. It is probable that he did not give the alarm until he found the acid working on him; at any rate, he walked from the table to the door, and unlocked it after taking the poison, called for assistance, and then walking to the sofa, stretched himself on it. *He had no convulsions.* Previous to the occurrence of the above cases, it has been held that, where prussic acid causes death slowly, convulsions come on after a notable interval, and where it acts speedily, no convulsions ensue, but death follows with such rapidity as to allow of none but the *simpleest* actions, and those performed with rapidity. From a review of the two cases extracted from the London Medical Gazette, we must allow the truth of the following inferences as to the action of hydrocyanic acid on the human body:

1st. Hydrocyanic acid is modified in its operation on the human frame, both as to time and phenomena, by the idiosyncrasy of the individual.

2d. That it *not unfrequently* is slow in manifesting its poisonous influence, allowing time for the performance of various complicated actions, and yet may destroy life without producing convulsions.

3d. That Judith Burwell could have performed the various actions attributed to her after swallowing the prussic acid, and have been found in the position stated by the witnesses in the trial of Freeman.—*British American Jour. of Medical and Physical Science.*

Montreal, September, 1847.

